# The transformation of Caribbean coral communities since humans

**DOI:** 10.1002/ece3.7808

**Published:** 2021-07-17

**Authors:** Katie L. Cramer, Mary K. Donovan, Jeremy B. C. Jackson, Benjamin J. Greenstein, Chelsea A. Korpanty, Geoffrey M. Cook, John M. Pandolfi

**Affiliations:** ^1^ Center for Biodiversity Outcomes and School of Life Sciences Arizona State University Tempe AZ USA; ^2^ Center for Global Discovery and Conservation Science and School of Geographical Sciences and Urban Planning Arizona State University Tempe AZ USA; ^3^ Center for Biodiversity and Conservation and Department of Paleontology American Museum of Natural History New York NY USA; ^4^ School of Social and Natural Sciences Roger Williams University Bristol RI USA; ^5^ MARUM Center for Marine Environmental Sciences University of Bremen Bremen Germany; ^6^ Department of Biology and Health Science New England College Henniker NH USA; ^7^ Centre for Marine Science School of Biological Sciences and ARC Centre of Excellence for Coral Reef Studies The University of Queensland St Lucia Qld Australia

**Keywords:** climate change, conservation paleobiology, coral reefs, fishing, historical ecology, pollution

## Abstract

The mass die‐off of Caribbean corals has transformed many of this region’s reefs to macroalgal‐dominated habitats since systematic monitoring began in the 1970s. Although attributed to a combination of local and global human stressors, the lack of long‐term data on Caribbean reef coral communities has prevented a clear understanding of the causes and consequences of coral declines. We integrated paleoecological, historical, and modern survey data to track the occurrence of major coral species and life‐history groups throughout the Caribbean from the prehuman period to the present. The regional loss of *Acropora* corals beginning by the 1960s from local human disturbances resulted in increases in the occurrence of formerly subdominant stress‐tolerant and weedy scleractinian corals and the competitive hydrozoan *Millepora* beginning in the 1970s and 1980s. These transformations have resulted in the homogenization of coral communities within individual countries. However, increases in stress‐tolerant and weedy corals have slowed or reversed since the 1980s and 1990s in tandem with intensified coral bleaching and disease. These patterns reveal the long history of increasingly stressful environmental conditions on Caribbean reefs that began with widespread local human disturbances and have recently culminated in the combined effects of local and global change.

## Graphical abstract

We integrated paleoecological, historical, and modern survey data to track the prevalence of major coral species and life‐history groups throughout the Caribbean from the prehuman period to present. This extended time series reveals that Caribbean coral communities have been transformed via three distinct stages since humans: (a) declines in competitive corals by the 1960s, (b) increases in stress‐tolerant and weedy corals occurring by the 1970s and 1980s, and (c) declines or leveling‐off of stress‐tolerant and weedy corals since the 1980s or 1990s. These patterns reveal the long history of increasingly stressful environmental conditions on Caribbean reefs that began with widespread local human disturbances and have recently culminated in the combined effects of local and global change.

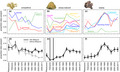

## 1 INTRODUCTION

Living cover of reef‐building corals has declined on Caribbean reefs by 50% to 80% since systematic monitoring began in the late 1970s (Gardner et al., 2003; Jackson et al., 2014) with many reefs altered from coral‐ to algal‐dominated habitats (Hughes et al., 2007; Jackson et al., 2014). Coral declines have been attributed to multiple anthropogenic stressors including fishing, land‐based sedimentation and pollution from agriculture and coastal development, and global warming as well as epizootics afflicting corals and urchins (Aronson & Precht, 2001; Hughes et al., 2007; Jackson et al., 2014). The mass mortality of the long‐spined sea urchin *Diadema antillarum* in the early 1980s removed the last abundant herbivore from reefs that were largely devoid of herbivorous fish after decades to centuries of overfishing (Jackson, 1997; Jackson et al., 2001; Lessios, Cubit, et al., 1984; Lessios, Robertson, et al., 1984; Pandolfi et al., 2003), precipitating an explosion of macroalgae on reefs across the Caribbean (Jackson et al., 2014). Outbreaks of White Band Disease appeared on many reefs around this same time, eventually killing over 80% of the remaining elkhorn coral *Acropora palmata* which previously dominated reef crest zones and staghorn coral *Acropora cervicornis* which previously dominated midslope zones (Bruckner, 2002; Bythell et al., 2004; Gladfelter, 1982). These events were followed by regional coral bleaching in the 1990s, leading to further increases in coral diseases and in some instances a further replacement of corals by macroalgae (Eakin et al., 2010). These factors acted synergistically to rapidly transform coral communities. A recent Caribbean‐wide analysis of *Acropora* presence and dominance from the prehuman period to the present revealed that the loss of these corals initially began in the 1950s and 1960s, decades before the first recorded observations of coral disease and bleaching (Cramer, Jackson et al., 2020). These changes are historically unprecedented—paleoecological studies show that modern Caribbean coral community composition, devoid of major framework building corals *Acropora* and *Orbicella* (Jackson et al., 2014), is unlike anything in the previous 220,000 years (Jackson, 1992; Mesolella, 1967; Pandolfi & Jackson, 2006).

While the long‐term decline of acroporid corals has been documented across the Caribbean, long‐term regional trends in the full reef coral community have not been assessed. Although isolated surveys of altered reefs have found an increase in the relative abundance of low relief “weedy” species such as *Porites* and *Agaricia* over the past few decades (Aronson et al., 2004, 2005; Cramer et al., 2012; Green et al., 2008), the magnitude and geographic extent of community change are unresolved. Reconstructing long‐term change in the full reef‐building coral community will shed light on the anthropogenic causes and ecological consequences of recent coral declines and can help guide management interventions by providing accurate baseline data from which to set appropriate recovery targets. We compiled an extensive dataset on the occurrence of common hermatypic coral species and genera at thousands of individual reef sites across the Caribbean spanning the Late Pleistocene epoch (~131,000 ybp)—when humans were absent from the Americas (Cooke, 2005)—up to 2011. By pinpointing the initial timing of major community transformations, we inferred general anthropogenic causes of change (i.e., local human stressors and/or climate change). To provide additional insight into the ecological context of change, we also tracked change in (a) major coral ecological guilds based on several life‐history traits and (b) inter‐site community heterogeneity.

## 2 METHODS

### 2.1 Coral community composition database

Data on coral species composition from the prehuman period to the present were compiled from semi‐quantitative, quantitative, and qualitative records from the primary peer‐reviewed scientific literature, government reports, and historical literature and were extracted from text, tables and figures, and maps. These data were collected via surveys of uplifted fossil reefs, analysis of coral fossils in reef matrix cores, qualitative field notes from boat‐based or snorkel surveys, high‐resolution aerial photographs and underwater field surveys using SCUBA. Most of the coral species composition data since the 1980s were received directly from contributors or gleaned from peer‐reviewed literature to construct the Global Coral Reef Monitoring Network (GCRMN) database that assessed trends in Caribbean reef benthic communities from 1970–2011 (Table S1; Jackson et al., 2014).

Temporal changes were tracked for 14 common coral taxonomic groups that were consistently recorded within each time bin (henceforth termed “species groups”: *A*. *cervicornis*, *A. palmata*, *Agaricia* spp., *Montastraea cavernosa*, *Colpophyllia* spp., *Pseudodiploria* spp.*, Madracis* spp., *Meandrina* spp., *Millepora* spp., *Orbicella* spp., branching *Porites* spp., *Porites astreoides*, *Siderastrea* spp., and *Stephanocoenia* spp.). To provide sufficient temporal resolution and ensure adequate sample sizes for assessing change over the full time series, data were grouped into eleven time bins: Late Pleistocene (~131,000–12,000 years ago, which encompasses the period prior to human settlement in the Caribbean region), Holocene (~9,100 years ago‐1500 AD, which encompasses the prehistoric period prior to European contact), 1500–1959, 1960–1969, 1970–1979, 1980–1984, 1985–1989, 1990–1994, 1995–2000, 2001–2004, and 2005–2011. Bins were reduced to 5‐year increments after 1980 (except for a 6‐year increment for the most recent bin) due to the large increase in reef survey effort following the mass die‐off of the urchin *D. antillarum*. The prehuman Pleistocene bin encompasses a period of high‐magnitude fluctuations in sea level and climate (during which *Acropora* coral dominance persisted in the Caribbean; Pandolfi & Jackson, 2006), whereas the prehistoric Holocene and subsequent bins encompass a period with higher stability in climate and sea level than the Pleistocene (Khan et al., 2017). Please see Table A1 for a timeline of major events affecting Caribbean reefs within each time bin.

Qualitative data were included in the database if, in addition to presence/absence information for at least one coral species, the following information was also available: (a) age of fossil data or year of observation of modern data, (b) original source of data, (c) country and island, coastline, or reef site, and (d) water depth or reef zone. Data were recorded at the survey level, with a survey constituting a unique combination of reef site, depth zone, and year. For surveys which included multiple replicates (i.e., transects or quadrats) at the same site, depth zone, and year/period, an overall value was computed for all replicates. Surveys constituted individual reef “sites” and in some cases encompassed more extensive areas such as entire reef tracts, bays, or banks.

We analyzed data from “reef crest” and “midslope” reef zones separately. Generally, the reef crest data spanned 0–6 m water depth and midslope data spanned between 6–20 m, as 6 m was the depth at which dominance typically transitioned from *A. palmata* to *A. cervicornis* in the semi‐quantitative and quantitative data. However, the reef crest/midslope zone delineation was made on a location‐by‐location basis by first considering water depth and, when available, additional environmental characteristics such as wave exposure and reef morphology. For some offshore locations with presumably higher water clarity, the cutoff was closer to 10 m. When a precise water depth was not available, we utilized *Acropora* species presence and/or dominance in addition to environmental characteristics to delineate between zones (Cramer, Jackson et al., 2020). When not reported in the papers from which data were extracted, paleo water depths were determined using the procedure outlined in Cramer, Jackson et al. (2020). Surveys from backreef habitats, reef flats, and reef pavements were excluded from our database, as these reef zones are not the preferred environments for Caribbean *Acropora* (Milliman, 1969).

Change in coral community composition was assessed using coral species presence/absence data, extracted from species rankings, presence/absence, percent living coral cover, number of individual colonies, and percent weight or volume of coral fossils. Only species included in the 14 commonly occurring species groups were considered in our analyses. Data recorded as “*Porites* spp.” were assumed to be branching *Porites* spp. because species within this complex are difficult to distinguish, were not consistently recognized as distinct until the 20th century (Jameson & Cairns, 2012), and were often recorded as a single species, “*P. porites*” in our database, whereas the morphologically distinct *Porites astreoides* was consistently recorded in our database and was therefore assigned to a separate category in our analyses. Data recorded as “*Montastraea* spp.” were assumed to be *Orbicella* spp. because species within this complex are difficult to distinguish and were not recognized as separate species until 1992 (Knowlton et al., 1992). The morphologically distinct *M. cavernosa* was consistently recorded in our database and was therefore assigned to a separate category in our analyses. *Pseudodiploria clivosa*, *Pseudodiploria strigosa*, and *Diploria labyrinthiformis* were assigned to *Pseudodiploria* spp., as these species were placed within the same genus until 2012 (Budd et al., 2012).

### 2.2 Coral life‐history strategies

To infer potential environmental changes driving coral community shifts, we also tracked trends in coral life‐history groups. We utilized a trait‐based classification approach that grouped scleractinians into four life‐history strategies separated primarily by colony morphology, growth rate, and reproductive mode (Darling et al., 2012). This configuration roughly follows Grime’s arrangement of plant species into three basic life‐history strategies: competitive species that maximize growth, stress‐tolerant species that maximize survival, and ruderal or weedy species that maximize fecundity (Grime, 1977, 2006). A fourth category, generalist species, represents a mixture of these strategies. However, because the generalist group was composed primarily of corals that are less common in the Caribbean and their presence and absence was not consistently noted in our dataset, this group was not included in our analyses. To add ecological context to these groupings, we collated from the literature taxon‐specific, qualitative measures of additional life‐history characteristics including sexual reproductive output (larval recruitment), asexual propagation via colony fragmentation, interspecific aggression, and susceptibility to disturbances such as sedimentation and bleaching (Table 1 and Table A2). Although larval recruitment represents the end‐point of fecundity, fertilization, dispersal, and early postsettlement mortality and is not a life‐history characteristic per se, this metric is closely linked to life‐history strategy (Smith, 1992) and provided valuable ecological insight into observed community change. Our life‐history groupings followed those determined via quantitative analyses in Darling et al. (2012) and Hardt (2007), with the addition of the hydrozoan *Millepora* described below. This addition was based on qualitative similarities in ranked values of life‐history traits described in Table 1; methods for assigning life‐history trait rankings are described in Table A2.

**TABLE 1:** Coral life‐history groups and their defining traits. Rankings compiled from data references listed in Table A2. Growth rate is average linear extension rate, reproductive output is larval recruitment rate. Growth rate ranking computed from mean of all published values separated into bottom/middle/top third percentiles of mean (e.g., 1–33, 34–66, 67–100 percentiles); ranges = 1.1–4.0, 5.0–7.0, and 13.2–119.5 mm/year for slow, moderate, and fast growth, respectively. Interspecific aggression ranking computed from experimental results from Lang (1973) and qualitative ranking from synthesis of literature, and rankings for all other traits computed from qualitative ranking from synthesis of literature

Life‐history group	Species group	Colony morphology	Growth rate (mm/year)	Reprod. mode	Reprod. output (sexual)	Asexual propagation	Interspecific aggression	Susceptibility to bleaching	Sediment rejection capacity/Sedimentation tolerance
Competitive	*Acropora cervicornis*	Large branching	Fast (119.5)	Spawner	Low	High	Moderate	High	Low
	*Acropora palmata*	Large branching	Fast (68.4)	Spawner	Low	High	Moderate	High	Low
	*(Millepora* spp.)	Plating and branching	Fast (13.2)	Spawner	Moderate	High	High	High	High
Stress‐tolerant	*Colpophyllia natans*	Domed	Moderate (7.0)	Spawner	Low	Low	High	Moderate to low	High
	*Pseudodiploria* spp.	Domed	Moderate (5.7)	Spawner	Low	Low	Moderate	Low	High
	*Meandrina* spp.	Domed	Slow (1.1)	Spawner	Low	Low	Moderate	Moderate	High
	*Montastrea cavernosa*	Domed	Moderate (5.8)	Spawner	Low	Low	Moderate	Low	High
	*Orbicella* spp.	Domed	Moderate (7.9)	Spawner	Low	Moderate to low	High	High	Moderate to high
	*Siderastrea* spp.	Domed	Slow (3.7)	Spawner (*s. Siderea*); brooder (*s. Radians*)	Low to moderate	Low	Low	Moderate to high	High
	*Stephanocoenia* spp.	Domed	Moderate (5.0)	Spawner	Low to moderate	Low	Low	High	High
Weedy	*Agaricia* spp.	Plating and foliose	Slow (1.6)	Brooder	High	Low	Low	High	Moderate
	Branching *Porites* spp.	Small branching	Fast (16.0)	Brooder	High	High	Low	Moderate to high	High
	*Porites astreoides*	Domed	Slow (4.0)	Brooder	High	Low	Low	High	High
	*Madracis* spp.	Small branching	Moderate (6.3)	Brooder	Moderate	High	Low	Low	High

The competitive life‐history group included *A. cervicornis, A. palmata,* and *Millepora* spp. This group is distinguished by fast growth rates, large branching morphologies that can outcompete other corals for light and/or space, medium to high levels of aggression, a spawning mode of reproduction but low rates of sexual recruitment, high propensity for asexual reproduction via fragmentation, and low tolerance to disturbances such as sedimentation and thermal stress (Table 1 and Table A2). This combination of traits historically allowed *Acropora* corals to dominate shallow, high‐energy reef environments prior to local and global anthropogenic stressors (Hughes & Jackson, 1985; Jackson, 1992; Pandolfi & Jackson, 2001). Although not included in the previous analyses of coral life‐history guilds, we included *Millepora* in the competitive category because of its *Acropora*‐like ability to preempt space on reefs due primarily to fragmentation and fast growth and its high susceptibility to bleaching (Table 1 and Table A2, Dubé, 2016; Loya et al., 2001).

The stress‐tolerant life‐history group includes *Colpophyllia natans*, *Pseudodiploria* spp., *Meandrina* spp., *Montastrea cavernosa*, *Orbicella* spp., *Siderastrea* spp., and *Stephanocoenia* spp. This group is distinguished by slow to moderate growth rates, large and domed morphologies with higher ability to clear sediment and other particles and resistant to storm damage, a spawning mode of reproduction with low to moderate sexual recruitment, low to high interspecific aggression, and relatively higher tolerance for sedimentation and thermal stress (Table 1 and Table A2). Although some stress‐tolerant corals have a higher susceptibility to bleaching, colony survival rates are typically high within this group (McClanahan & Muthiga, 1998). This combination of traits historically allowed these species to persist and dominate in environments subject to frequent, low‐magnitude disturbances such as sediment resuspension and temperature stress (Geister, 1977; Pandolfi & Jackson, 2001; Rutzler & Macintyre, 1982). Because the three extant *Orbicella* species were until recently classified as *Montastrea annularis* (Knowlton et al., 1992), we assigned *Orbicella* spp. to the stress‐tolerant category in accordance with the classification for *M. annularis* (Darling et al., 2012). However, this genera’s historical dominance on midslope zones on many Caribbean reefs and its high levels of interspecific aggression via mesenterial filaments (Lang, 1973) suggests this coral also has characteristics of the competitive life‐history group.

The weedy life‐history group includes *Agaricia* spp., *Madracis* spp., branching *Porites* spp., and *P. astreoides*. This group is distinguished by lower‐relief plating, foliose, branching, and domed morphologies with slow to fast growth rates, a brooding mode of reproduction that allows for rapid colonization at low population densities, generally high rates of sexual recruitment, high to low occurrence of asexual reproduction via fragmentation, low interspecific aggression, generally high susceptibility to bleaching, and high tolerance of sedimentation (Table 1 and Table A2). This combination of traits historically allowed these early‐successional species to opportunistically and rapidly colonize open spaces cleared by high‐magnitude acute disturbances (Bak & Engel, 1979; Hughes, 1985; Hughes & Jackson, 1985).

### 2.3 Analyses of coral community change

To estimate the proportion of reef sites containing each coral life‐history and species group in each time interval, we utilized binomial generalized linear mixed effects models that predicted the proportion of sites containing each life‐history group as a function of time bin, coral species group, and their interaction as fixed effects and country as a random effect. The interaction term was included to allow for varying temporal trends across individual taxonomic and functional groups. The random effect of country was included to account for uneven geographic sampling across time bins. To ensure equal numbers of surveys were included for each species group within a life‐history group, only surveys with presence/absence values for the entire complement of coral species within a given life‐history group were included in each model. Models were fitted using the *“glmer”* function in the R package “lme4.” Mean fitted values and 95% confidence intervals of the proportion of sites with a coral life‐history group and its constituent species groups were plotted for each time bin using the *“plot_model”* function in the R package “sjPlot.” Significant changes in mean fitted values relative to the Pleistocene baseline and subsequent peak values were assessed via a Tukey post hoc test using the *“emmeans”* function in the R package “emmeans.”

For the time series analyses, model performance was assessed via diagnostic plots of model residuals (quantile–quantile plots, pooled residuals vs. predicted values, and residuals of random and all significant fixed effects vs. predicted values) and via goodness‐of‐fit tests on pooled residuals (uniformity, outliers, and dispersion). Diagnostic plots and goodness‐of‐fit tests were produced for each model using the “DHARMa” package in R. Tests were carried out via a simulation‐based approach that transformed model residuals to a standardized scale. For each test, 1,000 simulations were conducted.

To assess the effects of coral community change on regional diversity patterns, we tracked temporal changes in community dissimilarity. We utilized species presence/absence matrices to compute Jaccard’s dissimilarity index (Jaccard, 1912). To account for the higher number of reef sites and countries added since the 1990s, we restricted our analysis to change in coral community dissimilarity within individual countries only. Within each time bin, the dissimilarity was computed between all possible combinations of reef sites located within the same country. For each country and time bin combination, a mean dissimilarity value was computed. To equalize the influence of each country and to avoid giving undue influence to countries with a larger number of surveys, mean dissimilarity values were computed for each country prior to computing the overall mean for a time bin. Uncertainty estimates were obtained via a bootstrap procedure that sampled with replacement from the distribution of mean within‐country dissimilarity values for each time bin *n* times (with *n* = number of countries for that bin). This resampling procedure was performed 1,000 times for each time bin; 95% confidence intervals were determined from the 5th and 95th quantiles of the resampled distributions. Significant differences in mean within‐country coral community dissimilarity values were determined by pairwise comparisons performed via permutation tests (with 1,000 iterations) using the *“pairwise.perm.t.test”* function in the *“RVAideMemoire”* package in R. All statistical analyses were performed using the program Rv3.4 (R Core Team, 2018), and all *p*‐value corrections for multiple testing were computed using the method outlined in Benjamini and Hochberg (1995).

## 3 RESULTS

### 3.1 Species community database

**FIGURE 1:**
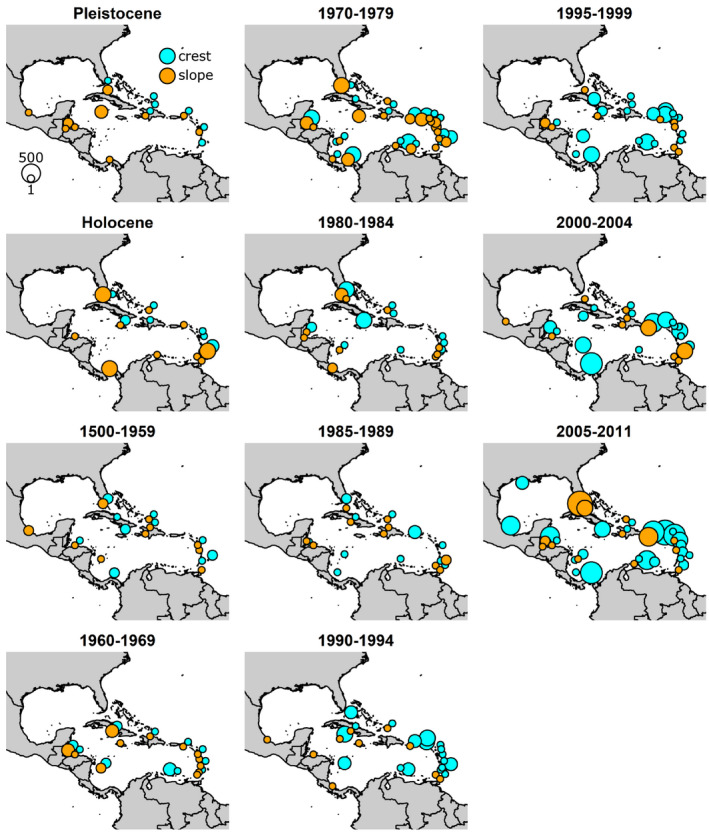
Distribution of presence/absence data for common Caribbean coral taxa. Size of circle proportional to total number of surveys across both reef zones and all bins combined (range = 1–541)

Coral species presence and absence data were compiled from 2,396 reef sites from 26 countries for the reef crest zone and 5,091 reef sites from 30 countries for the midslope zone (Figure 1). Full community data (containing a 0 or 1 value for all 14 common coral species groups) were available for all time periods and were compiled from 1,569 reef sites from 24 countries for the reef crest and 3,207 sites from 27 countries for the midslope zone (Tables A3 and A4).

### 3.2 Long‐term change in coral community composition

**FIGURE 2:**
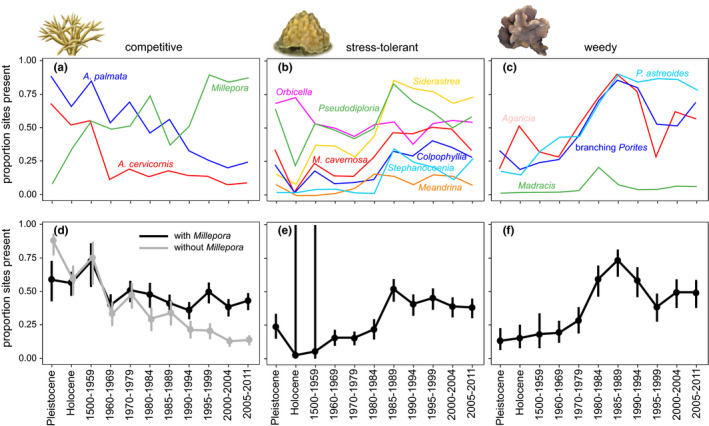
Occurrence of coral species (a‐c) and life history groups (d‐f) since the pre‐human period at reef crest zone. Vertical bars are 95% confidence intervals (not included for individual species for clarity of plot interpretation)

**FIGURE 3:**
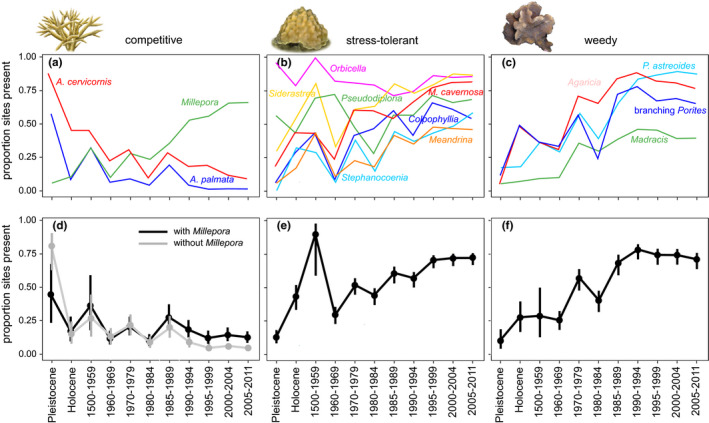
Occurrence of coral species (a‐c) and life history groups (d‐f) since the pre‐human period at midslope zone. Vertical bars are 95% confidence intervals (not included for individual species for clarity of plot interpretation)

Since the mid‐20th century, shallow water reefs across the Caribbean have transformed from systems dominated by competitive corals to systems dominated by stress‐tolerant and weedy corals (Figures 2 and 3). At both the reef crest and midslope zones, coral community change occurred via three stages: (a) significant declines in competitive corals relative to the prehuman baseline occurring by the 1960s, (b) significant increases in stress‐tolerant and weedy corals occurring by the 1970s and 1980s, and (c) significant declines or leveling‐off of stress‐tolerant and weedy corals since the 1980s or 1990s (Table 2, Figures 2 and 3). These trends were consistent whether *Millepora* was included or excluded from the competitive life‐history group (Figures 2 and 3). For competitive corals in both zones and stress‐tolerant and weedy corals in the midslope zone, the first significant change relative to the prehuman baseline occurred in the Holocene, reflecting contrasting environmental conditions and/or data types between the Pleistocene and Holocene (Figures 2 and 3).

**TABLE 2:** Trends in occurrence of coral life‐history and taxonomic groups. Trends deemed significant at the *p* < .05 level

Zone	Life‐history group	Taxon	Overall change (* = sig)	Earliest sig. change relative to Pleistocene	Peak in occurrence	Earliest sig. decline from peak
Crest	Competitive	(without *Millepora*) (with *Millepora*)	87%–13%* 54%–38%*	Holocene 1960–1969	Pleistocene 1500–1959	Holocene 1960–1969
		*Acropora cervicornis*	69%–9%*	1960–1969	Pleistocene	1960–1969
		*Acropora palmata*	89%–24%*	Holocene	Pleistocene	Holocene
		*Millepora* spp.	8%–88%*	1500–1959	1995–1999	–
	Stress‐tolerant		22%–37%*	1985–1989	1985–1989	1990–1994
		*Colpophyllia natans*	23%–28%	–	1995–1999	2005–2011
		*Pseudodiploria* spp.	65%–58%	Holocene	1985–1989	1995–1999
		*Meandrina* spp.	8%–7%	–	1980–1984	–
		*Montastrea cavernosa*	34%–33%	Holocene	1995–1999	–
		*Orbicella* spp.	69%–55%	1990–1994	Pleistocene	1990–1994
		*Siderastrea* spp.	15%–73%*	1980–1984	1985–1989	2000–2004
		*Stephanocoenia* spp.	2%–27%	1985–1989	1985–1989	1995–1999
	Weedy		1%–48%*	–	1985–1989	1990–1994
		*Agaricia* spp.	20%–57%*	1980–1984	1985–1989	1995–1999
		branching *Porites* spp.	33%–69%*	1970–1979	1985–1989	1995–1999
		*Madracis* spp.	>1%–6%	–	1980–1984	1985–1989
		*Porites astreoides*	17%–78%*	1960–1969	1985–1989	–
Slope	Competitive	(without *Millepora*) (with *Millepora*)	80%–4%* 44%–12%*	Holocene 1960–1969	Pleistocene 1500–1959	Holocene Holocene
		*Acropora cervicornis*	87%–8%*	Holocene	Precontact	Holocene
		*Acropora palmata*	58%–1%*	Holocene	1500–1959	Holocene
		*Millepora* spp.	5%–66%*	1995–1999	2005–2011	N/A
	Stress‐tolerant		8%–71%	Holocene	1500–1959	–
		*Colpophyllia natans*	6%–55%*	1970–1979	1995–1999	2005–2011
		*Pseudodiploria* spp.	57%–68%	1980–1984	1960–1969	–
		*Meandrina* spp.	6%–43%*	1985–1989	1995–1999	–
		*Montastrea cavernosa*	19%–82%*	1970–1979	2005–2011	N/A
		*Orbicella* spp.	96%–86%	1985–1989	1500–1959	–
		*Siderastrea* spp.	30%–87%*	1980–1984	2000–2004	–
		*Stephanocoenia* spp.	1%–59%*	Holocene	2005–2011	N/A
	Weedy		9%–70%*	1960–1969	1990–1994	decline
		*Agaricia* spp.	6%–77%*	Holocene	1990–1994	2005–2011
		Branching *Porites* spp.	12%–66%*	1970–1979	1990–1994	1995–1999
		*Madracis* spp.	6%–40%*	1970–1979	1990–1994	–
		*Porites astreoides*	18%–87%*	1970–1979	2000–2004	–

The assessment of trends for individual species groups revealed that at both reef zones, the occurrence of competitive *A. palmata* and *A. cervicornis* declined significantly between the Pleistocene and Holocene periods. After the Holocene, the next significant decline in competitive *Acropora* corals occurred in the 1960s; the occurrence of these corals remained significantly lower than prehuman levels from this point forward (Table 2, Figures 2a and 3a). In contrast, occurrence of the competitive hydrozoan *Millepora* increased significantly across the full time series, peaking in the late 1990s (crest) or early 2000s (midslope). At the reef crest zone, *Siderastrea* was the only stress‐tolerant taxon that increased significantly from the prehuman period to present, while at the midslope zone all stress‐tolerant taxa except for *Pseudodiploria* and *Orbicella* increased significantly across the full time series (Table 2, Figures 2b and 3b). The post‐1980s declines in the occurrence of stress‐tolerant corals that occurred at the reef crest zone were driven by declines in *Colpophyllia, Pseudodiploria, Montastrea cavernosa*, and *Siderastrea* while post‐1990s leveling‐off in the occurrence of this group at the midslope zone was driven by declining or consistent occurrence of *Colpophyllia,* *Pseudodiploria,* and *Montastrea cavernosa*. At both reef zones, all weedy taxa increased significantly across the full time series with the exception of *Madracis* in the reef crest (Table 2, Figures 2c and 3c). Initial significant increases in individual weedy coral species primarily occurred in the 1980s (crest) and in the 1970s (midslope). Significant post‐1980s declines in the weedy group at the reef crest reflect contemporaneous declines in *Agaricia*, branching *Porites*, and *P. astreoides*, while significant post‐1990s declines in the weedy group at the midslope reflect declines in *Agaricia* and branching *Porites*.

### 3.3 Community dissimilarity

**FIGURE 4:**
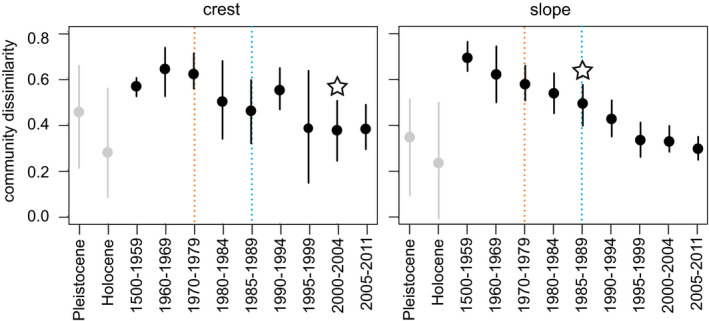
Within‐country dissimilarity of Caribbean coral communities since the prehuman period. Horizontal lines are 95% confidence intervals. Orange lines indicate first‐documented instances of White Band Disease; blue lines indicate first‐documented instances of widespread coral bleaching and the period following the die‐off of keystone herbivore urchin *Diadema antillarum*. Stars indicate earliest significant change relative to baseline period 1500–1959

Within‐country community dissimilarity declined significantly from the historic period to present at both reef zones; the decline was more striking in the latter (Figure 4). Because the Pleistocene and Holocene communities at both reef zones were more variable and had notably lower average dissimilarity values compared to subsequent periods—likely due to time‐averaging of the fossil data which comprise these time bins—we designated the historical time bin (1500–1959) as the baseline for this community metric. At both the reef crest and midslope zones, pairwise comparisons of mean within‐country dissimilarity values showed significant declines from the baseline historical period (1500–1959) to most recent period (2005–2011), with the first significant decline relative to the baseline occurring in 2000–2004 at the reef crest and in 1985–1989 at the midslope zones (Figure 4).

## 4 DISCUSSION

### 4.1 The transformation of coral communities from prehuman period to present

Our 131,000‐year record of coral community composition reveals that since humans arrived in the Caribbean, coral reefs throughout this region have transformed from systems dominated by competitive *Acropora* corals typified by fast growth, large and structurally complex colonies, high rates of reproduction via fragmentation, and lower tolerance to human disturbances to systems dominated by stress‐tolerant and weedy scleractinian corals with relatively slower growth, lower‐relief colony forms, and higher tolerance to human disturbances (Figures 2 and 3). Initial significant declines in *Acropora* occurred by the 1960s, a decade before the first recorded instances of White Band Disease and two decades before the die‐off of the *Diadema* urchin and widespread coral bleaching. From the prehuman period to the time of the first incidence of White Band Disease in the 1970s, *A. palmata* occurrence had already declined from ~90%–65% of sites at the reef crest and *A. cervicornis* occurrence had already declined from ~85%–30% of sites at the midslope zone (Figures 3 and 4). These early *Acropora* declines were related to local human stressors but not regional stressors including climate change (Cramer, Jackson et al., 2020), although more recent outbreaks of White Band Disease since the 1990s have been linked to climate change (Randall & van Woesik, 2015). The initial mid‐20th century loss of *Acropora* was followed by a lagged increase in the occurrence of stress‐tolerant and weedy corals that began in the 1970s and 1980s. By 2005–2011, these community shifts culminated in previously subdominant stress‐tolerant and weedy coral taxa being present at 2–3 times more sites than *Acropora*, the genus which had continuously dominated shallow reef zones across the Caribbean throughout the Pleistocene and Holocene up until the mid‐20th century (Cramer, Jackson et al., 2020; Jackson, 1992; Pandolfi & Jackson, 2006). The loss of *Acropora* also appears to have facilitated an increase in the occurrence of *Millepora*, a nonframework building hydrozoan with a competitive life‐history strategy (Figures 2a and 3a, Table A2).

Our time series suggests that the Caribbean‐wide loss of *Acropora* corals opened up both physical substrate and niche space for formerly subdominant corals with stress‐tolerant and weedy life‐history strategies and *Millepora* to occupy. The uniform rarity of *Acropora* and consistently high occurrence of weedy and stress‐tolerant species (particularly *Siderastrea*, *Agaricia*, and *Porites*) have greatly reduced the distinctiveness of modern coral communities at larger geographic scales, resulting in a Caribbean‐wide homogenization beginning in the 1960s at the midslope zone and in the 1980s at the reef crest zone (Figures 2, 3, 4). The homogenization of reef coral communities following the loss of *Acropora* and *Orbicella* has been reported for the Florida reef tract (Burman et al., 2012), Mexico (Estrada‐Saldívar et al., 2019), Belize and Panama (Aronson et al., 2005). However, our study confirms that coral communities have become more homogeneous across the entirety of the Caribbean. Although overall community distinctiveness has declined since the baseline historical period (1500–1959), this trend leveled off by the 1990s as increases in the occurrence of stress‐tolerant and weedy corals were halted following climate change‐related bleaching and disease (Figure 4).

Although broadly similar compositional changes occurred in coral communities at the reef crest and midslope zones, subtle distinctions in the trajectories of change between zones provide insight into their varying susceptibilities to different stressors. Declines in community dissimilarity were more striking at the midslope, reflecting the more precipitous decline of *A. cervicornis* at this zone compared to the decline of *A. palmata* at the reef crest (Figures 2a and 3a). This pattern may reflect declines in reef water quality from runoff, as increased turbidity would most likely have a greater effect on deeper reef zones due to increasing light attenuation with depth. However, the significant post‐1980s declines in the occurrence in stress‐tolerant and weedy corals that occurred at the reef crest (but not midslope) zone may reflect higher coral mortality from (a) anthropogenic bleaching in shallower zones that experience greater thermal stress (Baird et al., 2018; Bridge et al., 2013) and/or (b) the die‐off of the *Diadema* urchin that prefers shallower reef zones (Lessios, Cubit, et al., 1984; Lessios, Robertson, et al., 1984).

### 4.2 The role of local and global human stressors

Although climate change is currently imperiling reef ecosystems globally (Hughes et al., 2018), the early timing of the initial declines in Caribbean *Acropora* corals suggests that climate change was not responsible for this first phase of Caribbean coral community transformation. In the Caribbean, anthropogenic ocean warming did not become significant until the 1970s (Knutson et al., 2006; Sheppard & Rioja‐Nieto, 2005), and warming‐related coral bleaching was not observed until the late 1980s (Glynn, 1993). Our recent analysis of long‐term trends in the dominance of *A. cervicornis* and *A. palmata* showed that initial declines in the 1950s and 1960s were unrelated to regional or global stressors (i.e., anthropogenic temperature stress or hurricane exposure; Cramer, Jackson et al., 2020).

Instead, the early timing of initial changes in Caribbean coral communities implicates long‐standing local stressors such as fishing and land‐based pollution. However, the paucity of long‐term data on fishing effort/reef fish abundance or reef water quality precludes a quantitative assessment of the role of these activities in recent Caribbean‐wide reef ecosystem change. Consequently, despite the well‐established relationships between hermatypic coral persistence and abundant herbivorous reef fish populations and low‐sediment, low‐nutrient waters (Cramer et al., 2017; Fabricius, 2005; Hughes et al., 2007; Randall, 1961), historical fishing, and land clearing have been largely ignored in most analyses of Caribbean coral declines (Abelson, 2019). Fortunately, a few longer‐term datasets on water quality at various Caribbean reefs provide valuable insights into the role of land‐based runoff in coral community change. An analysis of seawater and macroalgae nitrogen content since the 1990s from the Florida Keys implicates land‐based nutrients from agriculture and development in the decades‐long coral declines within that reef tract (Lapointe et al., 2019). Studies based on historical and paleontological data also suggest that early reef ecosystem declines in Barbados and Panama may be attributed to increases in coastal runoff from historical land clearing for agriculture (Cramer et al., 2012; Cramer, O'Dea et al., 2020; Lewis, 1984). Last, the increase at both reef zones in the occurrence of *Millepora* and *Siderastrea*, corals that are particularly tolerant of high sedimentation and high turbidity conditions (Loya, 1976; de Weerdt, 1981), strongly indicates that declining water quality is a major driver of coral community change across the Caribbean.

Although initial declines in *Acropora* predate regional disturbances, subsequent changes bear a clear imprint of anthropogenic climate change. For instance, while the occurrence of stress‐tolerant and weedy corals is significantly higher today than the prehuman period, increases generally leveled off or reversed beginning in the late 1980s. The slowdown of increases in these corals is likely a response to the rapid explosion in benthic macroalgae following the die‐off of the keystone herbivore *D. antillarum* (Jackson et al., 2014) and increases in bleaching‐related mortality from anthropogenic temperature stress (Eakin et al., 2010). The more marked declines in *Agarcia* compared to branching *Porites* and *P. astreoides* at the reef crest likely reflect the relatively higher sensitivity of *Agaricia* to thermal stress: *Agaricia* experienced widespread bleaching episodes during thermal anomalies in the 1980s and 1990s (Aronson et al., 2000; Gates, 1990; Lasker et al., 1984). The post‐1980s declines/plateaus in stress‐tolerant and weedy species shown in this study are also a reflection of the increasingly frequent epizootics affecting these corals over the past 2–3 decades and that are linked to a combination of local and global anthropogenic stressors (Vega Thurber et al., 2020).

In contrast to the early transformation of Caribbean coral communities following the initial loss of *Acropora* in the 1950s/1960s, more recent changes since the 1980s/1990s demonstrate the heightened effects of local stressors and climate change acting on reefs simultaneously. Although our study suggests that White Band Disease was not the cause of initial *Acropora* declines, it confirms that it has unequivocally contributed to the loss of this genus: the second significant *Acropora* decline observed in our time series in the early 1980s immediately followed the first instances of this disease reported in the late 1970s (Gladfelter, 1982). Land‐based runoff has been shown to exacerbate coral bleaching and disease (Bruno et al., 2003; Lapointe et al., 2019; Wiedenmann et al., 2013), suggesting that reef eutrophication played a role in the emergence of these morbidities. Similarly, the region‐wide plateaus/declines in stress‐tolerant and weedy corals we observed since the 1980s/1990s reveal that local and global stressors are making Caribbean reef environments less suitable for those corals with the hardiest of life‐history strategies. Indeed, recent monitoring efforts have documented declines in several stress‐tolerant taxa from bleaching and disease that were initiated two decades ago (Edmunds & Elahi, 2007; Harvell et al., 2007) and show that several stress‐tolerant species are currently rapidly succumbing to the highly lethal Stony Coral Tissue Loss Disease that does not affect *Acropora* (Precht et al., 2016; Weil et al., 2009; van Woesik & Randall, 2017). Monitoring efforts are also documenting declines in weedy corals such as *Agaricia* due to recent Caribbean‐wide bleaching events (Walton et al., 2018). Finally, thermal stress and algal overgrowth are causing recruitment failure in Caribbean coral species regardless of life‐history guild (Arnold et al., 2010; Hughes & Tanner, 2000; Randall & Szmant, 2009). Thus, the shifts documented in our 131,000‐year record indicate a long history of increasingly stressful environmental conditions on Caribbean reefs that began with local human disturbances and have culminated in the combined effects of local and global change.

### 4.3 Challenges with assessing long‐term trends

**FIGURE A1:**
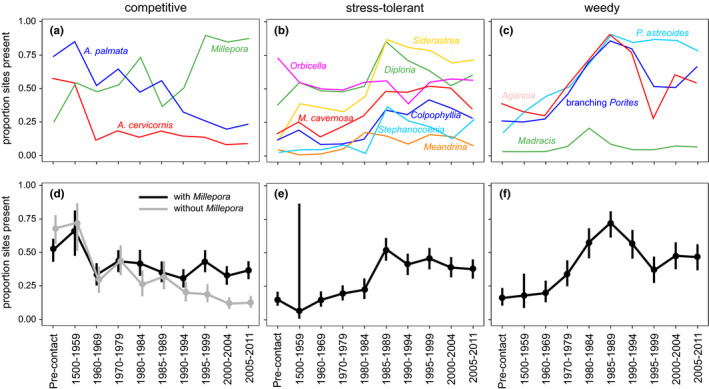
Occurrence of coral life‐history and species groups since humans at reef crest zone, with Pleistocene and Holocene combined into “precontact” time bin

**FIGURE A2:**
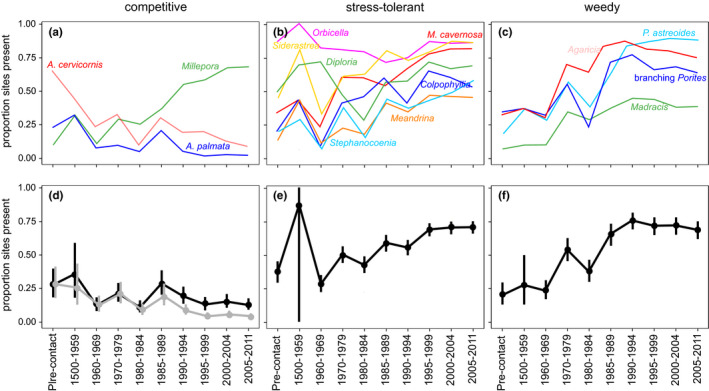
Occurrence of coral life‐history and species groups since humans at midslope zone, with Pleistocene and Holocene combined into “precontact” time bin

To track Caribbean coral community change prior to and since the arrival of humans, we used data from multiple sources, including uplifted fossil reefs, reef matrix cores, qualitative historical data, and underwater survey data, which may have led to uneven detection of particular coral taxa across different data types. For example, we observed conspicuously lower within‐country community dissimilarity observed within the fossil versus nonfossil time bins, likely due to the greater time‐averaging within the former (Figure 4). We also found significant differences in the occurrence of multiple coral taxa between the Pleistocene and Holocene, including declining occurrence of *A. cervicornis* at the midslope zone and increased occurrence of a number of stress‐tolerant and weedy taxa at both zones (Table 2, Figures 2 and 3). These differences could reflect declining rates of sea level rise during the late Holocene, which would favor increased dominance of more slowly growing species with massive colony forms (Hongo, 2012)—a trend we observe in our data from the midslope zone (Figure 3b). The relatively low occurrence of several species with massive colony growth forms in the Holocene period could also be due to the difficulty of sampling these colony types in the narrow‐diameter Holocene reef matrix cores that comprise much of the data from this time bin. Lower *A. cervicornis* occurrence in the Holocene compared to the Pleistocene could also be a result of inaccurate paleodepth estimates and/or underestimation of *A. cervicornis* abundance from the Holocene cores. However, *A. palmata* dominance increased slightly at the reef crest between the Pleistocene and Holocene, demonstrating that there was no bias against sampling this coral in the Holocene reef cores (Cramer, Jackson et al., 2020). Despite these discrepancies between the fossil time bins, Pleistocene coral communities were generally similar to those in the historical and early modern period (Figures 2 and 3), in agreement with other studies from the Caribbean Sea that showed remarkable comparability in Pleistocene and modern coral communities despite variation in growth rates among coral species, the higher degree of time‐averaging in fossil assemblages, and possible transport and mixing of fossil material (Jackson, 1992; Pandolfi & Jackson, 2001). Importantly, when data from the Pleistocene and Holocene periods are combined, overall trends in occurrence of coral functional and species groups are largely identical to those with these time periods separated: Declines in *Acropora* occurrence first occurred in the 1960s, followed by increases in stress‐tolerant and weedy species in the 1970s and 1980s (Table A5, Figures A1 and A2).

Although abundance data allow for a more robust assessment of community composition than presence/absence data, the exceptionally broad temporal, taxonomic, and geographic scales covered in this study necessitated the utilization of the latter. We recognize that occurrence does not equal abundance; although we found that the current occurrence of stress‐tolerant and weedy corals is higher than that from the prehuman and historical periods, the total abundance of living coral has declined by 50%–80% across the Caribbean since the initiation of quantitative surveys in the 1970s (Gardner et al., 2003; Jackson et al., 2014). However, ecological studies show that, on large spatial scales, *trends* in occurrence (proportion of sites occupied) are correlated with *trends* in abundance (MacKenzie et al., 2005; Weber et al., 2004), suggesting that the long‐term trends shown in this study are reliable proxies of qualitative trends in relative abundance. The occurrence trends observed in this study also correspond to recent trends in absolute abundance: The increasing occurrence of some stress‐tolerant and weedy corals (*Agaricia*, *P. astreoides*, branching *Porites*) over the past four decades in the midslope zone corresponds to trends in percent living cover from modern localized surveys of Caribbean coral communities (Green et al., 2008, Estrada‐Saldívar et al., 2019, González‐Barrios et al., 2021).

### 4.4 Conservation implications

The anthropogenic transformation of Caribbean coral communities into their novel configuration has widespread consequences for reef ecosystem functioning. First, the loss of competitive *Acropora* and stress‐tolerant *Orbicella* corals represents a massive simplification of reef architectural structure and loss of carbonate production that will likely compromise the ability of Caribbean reefs to keep pace with anthropogenic sea level rise (Alvarez‐Filip et al., 2009; Perry et al., 2014). Second, the loss of habitat complexity has the potential to reduce the diversity, biomass, and abundance of reef fish communities, the fisheries productivity of reefs, and the diversity of coral‐associated invertebrates (Cramer et al., 2012; Paddack et al., 2009; Richardson et al., 2018; Rogers et al., 2014). Third, coral community turnover has reduced the recovery potential of these reefs by selectively removing coral species with a spawning mode of reproduction and high larval dispersal rates (*Acropora* and *Orbicella*) and replacing them with species with a brooding mode of reproduction and low larval dispersal rates (*Agaricia* and *Porites*), limiting the ability of relatively intact reefs to re‐seed degraded ones (Knowlton, 2001). Although now‐dominant weedy corals such as *Porites* and *Agaricia* may still provide a reduced level of ecological benefits such as fine‐scale habitat complexity, protection from bioerosion of reef framework, and sediment and rubble production (González‐Barrios et al., 2021), the hastening decline of even these hardiest of species over the most recent decades is further diminishing the geo‐ecological functioning of Caribbean reefs. As climate change impacts accelerate on Caribbean reefs and exacerbate the effects of long‐standing local human disturbances, the simultaneous mitigation of both local and global stressors is the only viable path to reef persistence. Indeed, recent studies from the Caribbean and Great Barrier Reef highlight the prospect of enhanced reef resiliency (but not resistance) to climate change impacts when land‐based nutrification and overfishing are alleviated (Lapointe et al., 2019; MacNeil et al., 2019; Mellin et al., 2016; Steneck et al., 2019).

## CONFLICT OF INTEREST

The authors declare that they have no competing interests.

## AUTHOR CONTRIBUTIONS

**Katie Cramer:** Conceptualization (lead); Data curation (lead); Formal analysis (lead); Methodology (lead); Project administration (equal); Software (lead); Visualization (lead); Writing‐original draft (lead); Writing‐review & editing (equal). **Mary Donovan:** Data curation (equal); Formal analysis (supporting); Methodology (supporting); Writing‐review & editing (equal). **Jeremy Jackson:** Conceptualization (equal); Funding acquisition (lead); Project administration (lead); Writing‐review & editing (equal). **Benjamin Greenstein:** Conceptualization (equal); Data curation (equal); Methodology (equal); Writing‐review & editing (equal). **Chelsea Korpanty:** Data curation (equal); Writing‐review & editing (equal). **Geoffrey Cook:** Data curation (equal); Writing‐review & editing (equal). **John M. Pandolfi:** Conceptualization (equal); Data curation (equal); Methodology (equal); Writing‐review & editing (equal).

## Supporting information

Table S1

## Data Availability

All data have been deposited on Dryad (https://doi.org/10.5061/dryad.ht76hdrg0).

## 1

**TABLE A1:** Time bins included in the analysis of long‐term change in Caribbean coral communities and significant events affecting reef environments and detection of ecological change by researchers. Timeline sourced from Jackson et al. (2014) and Cramer, Jackson et al. (2020)

Time bin	Significant events
Pleistocene (131,000–12,000 years ago)	Period prior to human settlement in Caribbean; high‐magnitude fluctuations in sea level and climate with transitions from glacial to interglacial periods
Holocene (9,100 years ago–1500 AD)	Humans settle in Caribbean; sea‐level rise (following last glacial period) slows and sea level begins to stabilize; first European contact in Caribbean
1500–1959	Increasing utilization of reef resources by European colonizers; decline of indigenous populations from genocide and disease; first application of synthetic pesticides and fertilizers in Caribbean watersheds in 1950s; first widespread application of synthetic pesticides on agricultural crops in the Caribbean in 1950s
1960–1969	Increase in fertilizer use and pesticide imports in Caribbean region
1970–1979	First recorded incidence of White Band Disease in Caribbean *Acropora* corals; first signal of anthropogenic ocean warming in Caribbean; first systematic monitoring of Caribbean reefs
1980–1984	Mass die‐off of urchin *Diadema antillarum* due to disease in 1982–1983; increase in macroalgae on many Caribbean reefs
1985–1989	First warming‐related coral bleaching outbreaks
1990–1994	First regional‐scale coral bleaching in Caribbean; increase in coral disease outbreaks; intensification of region‐wide reef monitoring programs
1995–1999	Extreme heating event resulting in mass coral bleaching in 1998
2000–2004	Massive loss of reef architectural complexity following coral bleaching in 1999
2005–2011	Extreme heating events and coral bleaching outbreaks in 2005 and 2010

**TABLE A2:** References utilized to calculate rankings for various coral life‐history traits

Life‐history group	Species group	Colony morph.	Growth rate	Reprod. mode	Reprod. output (sexual)	Asexual prop.	Interspp. aggression	Bleaching suscept.	Sediment rejection capacity/Sedimentation tolerance
Competitive	*Acropora cervicornis*	1	3–6	26	27–29	39, 40	44	47, 48	52, 53
	*Acropora palmata*	1	3–5, 7	26	27, 29	31, 40	44	47, 48	52, 53
	(*Millepora* spp.)	2	8–12	2	30, 31	41	45, 46	49, 47, 48	54
Stress‐tolerant	*Colpophyllia natans*	1	4, 5	26	30, 32	40	44	48, 50	52
	*Pseudodiploria* spp.	1	4, 5, 13	26	27, 34, 35, 36	40	44	48	52, 53
	*Meandrina* spp.	1	4	26	31, 32	40	44	48	52
	*Montastrea cavernosa*	1	4, 5	26	33, 37	40	44	48	52, 55
	*Orbicella* spp.	1	4, 5, 14–20	26	27, 33, 35	40, 42, 43	44	51	52, 53
	*Siderastrea* spp.	1	4, 5, 21	26	30, 33, 34	40	44	48	52, 53, 56, 57, 58, 59
	*Stephanocoenia* spp.	1	22	26	31	40	44	48	52, 58
Weedy	*Agaricia* spp.	1	4, 5	26	31, 33, 35, 36, 38	33	44	48	52
	Branching *Porites* spp.	1	5	26	35, 36	40, 33	44	48, 50	52, 53
	*Porites astreoides*	1	3–5, 13, 14, 23	26	31, 34, 35, 36	40	44	48	52, 53
	*Madracis* spp.	1	24, 25	26	30, 31, 33	31, 40, 33	44	48	52

**TABLE A3:** Number of reef sites with presence/absence data for all 14 common coral taxa at reef crest zone

Country	Pleist.	Holo.	1500–1959	1960–1969	1970–1979	1980–1984	1985–1989	1990–1994	1995–1999	2000–2004	2005–2011
Antigua & Barbuda	1										2
Bahamas	1				2				3		3
Barbados	6		2	1	3	22	5		1	27	
Belize	2	9		7	8	1	1	1	5	4	
British Virgin Islands					3						
Cayman Islands	17			26	4		1				
Colombia				6	1	1		6		9	
Costa Rica						6					
Cuba				11				1	1		12
Dominican Republic	2	2		1	2						
Dutch Caribbean	1			4	6		1			3	7
Florida		3			10	13	1	51	95	180	268
Guadel. & Martinique		1			5		1			1	7
Grenada											4
Honduras							2				
Jamaica			7	1	4				4		9
Mexico	2	17	4	23	4	17	7	1	1	4	
Panama		4			8		93	101	95	56	89
Puerto Rico		10		5	3						15
St. Lucia					1						
St. Vinc. & Grenadines				2	2						
Trinidad & Tobago						5					
US Virgin Islands		17	1		8	6	20	14		3	5
Venezuela				2	5				1		
Total	32	63	14	89	79	71	132	175	206	287	421

**TABLE A4:** Number of reef sites with presence/absence data for 14 common coral taxa at midslope zone

Country	Pleist.	Holo.	1500–1959	1960–1969	1970–1979	1980–1984	1985–1989	1990–1994	1995–1999	2000–2004	2005–2011
Bahamas	7	0	1	6	1			10		16	23
Barbados	7	1	3		7	2		11	2	8	4
Belize		12		6	19	3	1	1	52	18	53
British Virgin Islands					2			22	40	40	64
Cayman Islands				32	2		3	39	7	7	8
Colombia	1			5	2	2		4	14	46	7
Costa Rica						7		1			
Cuba				7				2	12		25
Dominica											9
Dominican Republic	2	2			4				2	1	
Dutch Caribbean		4		4	17	92		15	14	19	52
Florida	1	5	1		45	10	1	68	181	232	509
Guadel. & Martinique					3					27	48
Grenada					3						15
Haiti	2										
Honduras							2		2	1	1
Jamaica			2	2	16	9	16		8	4	26
Mexico		1		26	5	30	22	4		14	65
Nicaragua								2	3		
Panama					12		72	53	43	84	132
Puerto				1	6			8	13	13	66
St. Kitts & Nevis											2
St. Lucia					2						9
St. Vinc. & Grenadines					5						2
Turks & Caicos									1		
Trinidad & Tobago					1	10		1	4	5	6
U.S. Virgin Islands		3			5	16	6	22	15	101	164
Total	20	28	7	89	157	181	123	263	413	636	1290

**TABLE A5:** Trends in coral life‐history groups with Pleistocene and Holocene periods combined into a single “Precontact” time bin. Trends deemed significant at the *p* < .05 level

Zone	Life‐history group	Overall change (*sig)	Earliest sig. change relative to Pleistocene	Peak in occurrence
Crest	Competitive (without *Millepora*)	67–13%*	1960–1969	1500–1959
	Competitive (with *Millepora*)	51–38%*	1960–1969	1500–1959
	Stress‐tolerant	14–37%*	1985–1989	1985–1989
	Weedy	15–48%*	1970–1979	1985–1989
Midslope	Competitive (without *Millepora*)	28–4%*	1960–1969	Precontact
	Competitive (with *Millepora*)	27–12%*	1960–1969	1500–1959
	Stress‐tolerant	37–71%	1970–1979	2005–2011
	Weedy	20–70%*	1970–1979	1990–1994

**References for Table A2**

1. Darling, E. S., Alvarez‐Filip, L., Oliver, T. A., McClanahan, T. R., & Côté, I. M. (2012). Evaluating life‐history strategies of reef corals from species traits. Ecology Letters, 15(12), 1378–1386.
2. Lewis, J.B., 1989. The ecology of Millepora. Coral reefs, 8(3), pp.99–107.
3. Crabbe M.J.C. (2010). Topography and spatial arrangement of reef‐building corals on the fringing reefs of North Jamaica may influence their response to disturbance from bleaching. Mar. Environ. Res., 69, 158–162.
4. Crabbe M.J.C. (2009). Scleractinian coral population size structures and growth rates indicate coral resilience on the fringing reefs of North Jamaica. Mar. Environ. Res., 67, 189–198.
5. Huston M. (1985). Variation in coral growth rates with depth at Discovery Bay, Jamaica. Coral Reefs, 4, 19–25.
6. Torres J.L., Armstrong R.A., Corredor J.E. & Gilbes F. (2007). Physiological responses of Acropora cervicornis to increased solar irradiance. Photochem. Photobiol., 83, 839–850.
7. Bak R.P.M., Nieuland G. & Meesters E.H. (2009). Coral growth rates revisited after 31 years: What is causing lower extension rates in Acropora palmata? Bull. Mar. Sci., 84, 287–294.
8. Lewis, J.B., 1991. Banding, age and growth in the calcareous hydrozoan Millepora complanata Lamarck. Coral Reefs, 9(4), pp.209–214.
9. Edmunds, P.J., 1999. The role of colony morphology and substratum inclination in the success of Millepora alcicornis on shallow coral reefs. Coral Reefs, 18(2), pp.133–140.
10. Stromgren, T., 1976. Skeleton growth of the hydrocoral Millepora complanata Lamarck in relation to light. Limnology and Oceanography, 21(1), pp.156–160.
11. Weerdt WH de (1981) Transplantation experiments with Caribbean Millepora species (Hydrozoa, Coelenterata), including some ecological observations on growth forms. Bijdr Dierk 51:1–19
12. Witman JD (1988) Effects of predation by the fireworm Hermadice carunculata on Milleporid corals. Bull Mar Sci 42:446–458
13. Logan A. & Tomascik T. (1991). Extension growth rates in two coral species from high‐latitude reefs of Bermuda. Coral Reefs, 10, 155–160.
14. Carricart‐Ganivet J.P. (2004). Sea surface temperature and the growth of the West Atlantic reef‐building coral Montastraea annularis. J. Exp. Mar. Biol. Ecol., 302, 249–260.
15. Cruz‐Pinon G., Carricart‐Ganivet J.P. & Espinoza‐Avalos J. (2003). Monthly skeletal extension rates of the hermatypic corals Montastraea annularis and Montastraea faveolata: biological and environmental controls. Mar. Biol., 143, 491–500.
16. Mendes J. (2004). Timing of skeletal band formation in Montastraea annularis: Relationship to environmental and endogenous factors. Bull. Mar. Sci., 75, 423–437.
17. Mendes J.M. & Woodley J.D. (2002). Effect of the 1995‐1996 bleaching event on polyp tissue depth, growth, reproduction and skeletal band formation in Montastraea annularis. Mar. Ecol. Prog. Ser., 235, 93–102.
18. Tomascik T. & Sander F. (1985). Effects of eutrophication on reef‐building corals I. Growth rate of the reef‐building coral Montastrea annularis. Mar. Biol., 87, 143–155.
19. Saenger C., Cohen A.L., Oppo D.W. & Hubbard D. (2008). Interpreting sea surface temperature from strontium/calcium ratios in Montastrea corals: link with growth rate and implications for proxy reconstructions. Paleoceanography, 23, PA3102.
20. Helmle K.P., Dodge R.E., Swart P.K., Gledhill D.K. & Eakin C.M. (2011). Growth rates of Florida corals from 1937 to 1996 and their response to climate change. Nature Comm., 2, 215–6.
21. Castillo K.D., Ries J.B. & Weiss J.M. (2011). Declining coral skeletal extension for forereef colonies of Siderastrea siderea on the Mesoamerican barrier reef system, southern Belize. PLoS ONE, 6, e14615.
22. Moore W.S. & Krishnaswami S. (1972). Coral growth rates using 228Ra and 210Pb. Earth Planet. Sci. Lett., 15, 187–190.
23. Elizalde‐Rendón E.M., Horta‐Puga G., González‐Diaz P. & Carricart‐Ganivet J.P. (2010). Growth characteristics of the reef‐building coral Porites astreoides under different environmental conditions in the Western Atlantic. Coral Reefs, 29, 607–614.
24. Bruno, J.F. and Edmunds, P.J., 1997. Clonal variation for phenotypic plasticity in the coral Madracis mirabilis. Ecology, 78(7), pp.2177–2190.
25. Nagelkerken, I., Bouma, S., Van Den Akker, S. and Bak, R.P.M., 2000. Growth and survival of unattached Madracis mirabilis fragments transplanted to different reef sites, and the implication for reef rehabilitation. Bulletin of Marine Science, 66(2), pp.497–505.
26. Baird, A.H., Guest, J.R. and Willis, B.L., 2009. Systematic and biogeographical patterns in the reproductive biology of scleractinian corals. Annual Review of Ecology, Evolution, and Systematics, 40, pp.551–571.
27. Rylaarsdam, K.W., 1983. Life histories and abundance patterns of colonial corals on Jamaican reefs. Marine ecology progress series. Oldendorf, 13(2), pp.249–260.
28. Hughes, T.P. and Jackson, J.B.C., 1985. Population dynamics and life histories of foliaceous corals. Ecological Monographs, 55(2), pp.141–166.
29. Sammarco, P.P., 1985, January. The Great Barrier Reef vs. the Caribbean: comparisons of grazers, coral recruitment patterns and reef recovery. In Proceedings of the 5th International Coral Reef Congress, Tahiti, 27 May‐1 June 1985‐pages: 4: 391–398.
30. Dustan P., 1977. Vitality of reef coral populations off Key Largo, Florida: Recruitment and mortality. ‐Envir.‐ Geol. 2: 101–107.
31. Bak, R.P.M. and Engel, M.S., 1979. Distribution, abundance and survival of juvenile hermatypic corals (Scleractinia) and the importance of life history strategies in the parent coral community. Marine Biology, 54(4), pp.341–352.
32. Rogers, C.S., Fitz, H.C., Gilnack, M., Beets, J. and Hardin, J., 1984. Scleractinian coral recruitment patterns at salt river submarine canyon, St. Croix, US Virgin Islands. Coral Reefs, 3(2), pp.69–76.
33. Hughes, T.P., 1985. Life histories and population dynamics of early successional corals. Antenne Museum‐EPHE.
34. Smith, S.R. (1992) Patterns of coral recruitment and post settlement mortality on Bermudas reefs – comparisons to Caribbean and Pacific reefs. American Zoologist, 32, 663–673
35. Hughes, T.P. and Tanner, J.E., 2000. Recruitment failure, life histories, and long‐term decline of Caribbean corals. Ecology, 81(8), pp.2250–2263.
36. Carpenter, R.C. and Edmunds, P.J., 2006. Local and regional scale recovery of Diadema promotes recruitment of scleractinian corals. Ecology letters, 9(3), pp.271–280.
37. Irizarry‐Soto, E. and Weil, E., 2009. Spatial and temporal variability in juvenile coral densities, survivorship and recruitment in La Parguera, southwestern Puerto Rico. Caribbean Journal of Science, 45(2–3), pp.269–281.
38. Meyer, C. L., Birkeland, C. E. (1974). Marine studies. In: Rubinoff, R. W. (ed.) Environmental monitoring and baseline data compiled under the Smithsonian Institution Environmental Sciences program, Smithsonian Tropical Research Institution, Canal Zone, Balboa, p. 134–137, 242–250
39. Shinn, E. (1976). Coral reef recovery in Florida and the sian Gulf. Environ. Geol. 1: 241–254
40. Highsmith, R.C., 1982. Reproduction by fragmentation in corals. Marine ecology progress series. Oldendorf, 7(2), pp.207–226.
41. Lewis, J. B. Biology and ecology of the hydrocoral Millepora on coral reefs. Adv. Mar. Biol. 50, 1–55 (2006).
42. Jones, J. A. (1977). Morphology and development of south eastern Florida patch reefs. Proc. 3rd. Int. Coral Reef Symp. 2: 231–235
43. Foster, N.L., Baums, I.B. and Mumby, P.J., 2007. Sexual vs. asexual reproduction in an ecosystem engineer: the massive coral Montastraea annularis. Journal of Animal Ecology, 76(2), pp.384–391.
44. Lang J (1973) Interspecific aggression by scleractinian corals. 2. Why the race is not only to the swift. Bull Mar Sci 23:260–279
45. Wahle CM (1980) Detection, pursuit, and overgrowth of tropical gorgonians by milleporid hydrocorals: perseus and medusa revisited. Science 209:689–691
46. Wegener C, Martin B, Didden C, Edmunds PJ (2018) Overgrowth of Caribbean octocorals by milleporid hydrocorals. Invertebr Biol 137:29–37
47. Loya, Y., Sakai, K., Yamazato, K., Nakano, Y., Sambali, H. and Van Woesik, R., 2001. Coral bleaching: the winners and the losers. Ecology letters, 4(2), pp.122–131.
48. McClanahan, T.R., Weil, E., Cortés, J., Baird, A.H. and Ateweberhan, M., 2009. Consequences of coral bleaching for sessile reef organisms. In Coral bleaching (pp. 121–138). Springer, Berlin, Heidelberg.
49. Marshall, P.A. and Baird, A.H., 2000. Bleaching of corals on the Great Barrier Reef: differential susceptibilities among taxa. Coral Reefs, 19, pp.155–163.
50. Whelan, K.R.T., Miller, J., Sanchez, O. and Patterson, M., 2007. Impact of the 2005 coral bleaching event on Porites porites and Colpophyllia natans at Tektite Reef, US Virgin Islands. Coral Reefs, 26(3), pp.689–693.
51. Miller, J., Waara, R., Muller, E. and Rogers, C., 2006. Coral bleaching and disease combine to cause extensive mortality on reefs in US Virgin Islands. Coral Reefs, 25(3), p.418.
52. Bak, R.P.M. and Elgershuizen, J.H.B.W., 1976. Patterns of oil‐sediment rejection in corals. Marine Biology, 37(2), pp.105–113.
53. Erftemeijer, P.L. and Lewis III, R.R.R., 2006. Environmental impacts of dredging on seagrasses: a review. Marine pollution bulletin, 52(12), pp.1553–1572.
54. de Weerdt, W.H., 1981. Transplantation experiments with Caribbean Millepora species (Hydrozoa, Coelenterata), including some ecological observations on growth forms. Bijdragen tot de Dierkunde, 51(1), pp.1–19.
55. Lasker 1980. Sediment rejection by reef corals: The roles of behavior and morphology in Montastrea cavernosa (Linnaeus)
56. Loya, Y., 1976. Effects of water turbidity and sedimentation on the community structure of Puerto Rican corals. Bulletin of Marine Science, 26(4), pp.450–466.
57. Hubbard, J.A. and Pocock, Y.P., 1972. Sediment rejection by recent scleractinian corals: a key to palaeo‐environmental reconstruction. Geologische Rundschau, 61(2), pp.598–626.
58. Rice SA. 1984. Effects of suspended sediment and burial upon survival and growth of Eastern Gulf of Mexico Corals. Camp Dresser & McKee, Inc. Mote Marine Laboratory Technical Report no 87. 58 p.
59. Cortes, J., Risk, M.J., 1985. A reef under siltation stress: Cahuita, Costa Rica. Bulletinof Marine Science 36, 339–356.

## References

[ece37808-bib-0001] Abelson, A. (2019). Are we sacrificing the future of coral reefs on the altar of the “climate change” narrative? ICES Journal of Marine Science, 77(1), 40–45. 10.1093/icesjms/fsz226

[ece37808-bib-0002] Alvarez‐Filip, L. , Dulvy, N. K. , Gill, J. A. , Côté, I. M. , & Watkinson, A. R. (2009). Flattening of Caribbean coral reefs: Region‐wide declines in architectural complexity. Proceedings of the Royal Society B: Biological Sciences, 276(1669), 3019–3025. 10.1098/rspb.2009.0339 PMC2817220 19515663

[ece37808-bib-0003] Arnold, S. N. , Steneck, R. S. , & Mumby, P. J. (2010). Running the gauntlet: Inhibitory effects of algal turfs on the processes of coral recruitment. Marine Ecology Progress Series, 414, 91–105. 10.3354/meps08724

[ece37808-bib-0004] Aronson, R. B. , Macintyre, I. G. , Lewis, S. A. , & Hilbun, N. L. (2005). Emergent zonation and geographic convergence of coral reefs. Ecology, 86, 2586–2600. 10.1890/05-0045

[ece37808-bib-0005] Aronson, R. B. , Macintyre, I. G. , Wapnick, C. M. , & O'Neill, M. W. (2004). Phase shifts, alternative states, and the unprecedented convergence of two reef systems. Ecology, 85, 1876–1891. 10.1890/03-0108

[ece37808-bib-0006] Aronson, R. B. , & Precht, W. F. (2001). White‐band disease and the changing face of Caribbean coral reefs. Hydrobiologia, 460, 25–38.

[ece37808-bib-0007] Aronson, R. B. , Precht, W. F. , Macintyre, I. G. , & Murdoch, T. J. (2000). Ecosystems: Coral bleach‐out in Belize. Nature, 405, 36. 10.1038/35011132 10811207

[ece37808-bib-0008] Baird, A. H. , Madin, J. S. , Álvarez‐Noriega, M. , Fontoura, L. , Kerry, J. T. , Kuo, C. Y. , Precoda, K. , Torres‐Pulliza, D. , Woods, R. M. , Zawada, K. J. , & Hughes, T. P. (2018). A decline in bleaching suggests that depth can provide a refuge from global warming in most coral taxa. Marine Ecology Progress Series, 603, 257–264. 10.3354/meps12732

[ece37808-bib-0009] Bak, R. P. M. , & Engel, M. S. (1979). Distribution, abundance and survival of juvenile hermatypic corals (*Scleractinia*) and the importance of life history strategies in the parent coral community. Marine Biology, 54, 341–352. 10.1007/BF00395440

[ece37808-bib-0010] Benjamini, Y. , & Hochberg, Y. (1995). Controlling the false discovery rate: A practical and powerful approach to multiple testing. Journal of the Royal Statistical Society: Series B (Methodological), 57(1), 289–300. 10.1111/j.2517-6161.1995.tb02031.x

[ece37808-bib-0011] Bridge, T. C. , Hoey, A. S. , Campbell, S. J. , Muttaqin, E. , Rudi, E. , Fadli, N. , & Baird, A. H. (2013). Depth‐dependent mortality of reef corals following a severe bleaching event: implications for thermal refuges and population recovery. F1000Research, 2, 187. 10.12688/f1000research.2-187.v1 24627789 PMC3938179

[ece37808-bib-0012] Bruckner, A. W. (2002). Proceedings of the Caribbean Acropora workshop: Potential application of the U.S. Endangered Species Act as a conservation strategy. Silver Spring, MD: NOAA Technical Memorandum NMFS‐OPR‐24.

[ece37808-bib-0013] Bruno, J. F. , Petes, L. E. , Harvell, C. D. , & Hettinger, A. (2003). Nutrient enrichment can increase the severity of coral diseases. Ecology Letters, 6, 1056–1061.

[ece37808-bib-0014] Budd, A. F. , Fukami, H. , Smith, N. D. , & Knowlton, N. (2012). Taxonomic classification of the reef coral family Mussidae (Cnidaria: Anthozoa: Scleractinia). Zoological Journal of the Linnean Society, 166, 465–529.

[ece37808-bib-0015] Burman, S. G. , Aronson, R. B. , & van Woesik, R. (2012). Biotic homogenization of coral assemblages along the Florida reef tract. Marine Ecology Progress Series, 467, 89–96. 10.3354/meps09950

[ece37808-bib-0016] Bythell, J. , Pantos, O. , & Richardson, L. (2004). White plague, white band, and other “white” diseases. In E. Rosenberg , & Y. Loya (Eds.), Coral health and disease (pp. 351–365). Springer.

[ece37808-bib-0017] Cooke, R. (2005). Prehistory of native Americans on the Central American land bridge: Colonization, dispersal, and divergence. Journal of Archaeological Research, 13(2), 129–187. 10.1007/s10804-005-2486-4

[ece37808-bib-0018] Cramer, K. L. , Jackson, J. B. , Angioletti, C. V. , Leonard‐Pingel, J. , & Guilderson, T. P. (2012). Anthropogenic mortality on coral reefs in Caribbean Panama predates coral disease and bleaching. Ecology Letters, 15, 561–567. 22462739 10.1111/j.1461-0248.2012.01768.x

[ece37808-bib-0019] Cramer, K. L. , Jackson, J. B. , Donovan, M. K. , Greenstein, B. J. , Korpanty, C. A. , Cook, G. M. , & Pandolfi, J. M. (2020). Widespread loss of Caribbean acroporid corals was underway before coral bleaching and disease outbreaks. Science Advances, 6, eaax9395. 10.1126/sciadv.aax9395 32426458 PMC7176417

[ece37808-bib-0020] Cramer, K. L. , O’Dea, A. , Clark, T. R. , Zhao, J. , & Norris, R. D. (2017). Prehistorical and historical declines in Caribbean coral reef accretion rates driven by loss of parrotfish. Nature Communications, 8, 14160. 10.1038/ncomms14160 PMC5267576 28112169

[ece37808-bib-0021] Cramer, K. L. , O’Dea, A. , Leonard‐Pingel, J. S. , & Norris, R. D. (2020). Millennial‐scale change in Caribbean coral reef ecosystem structure and the role of human and natural disturbance. Ecography, 43(2), 283–293.

[ece37808-bib-0022] Darling, E. S. , Alvarez‐Filip, L. , Oliver, T. A. , McClanahan, T. R. , & Côté, I. M. (2012). Evaluating life‐history strategies of reef corals from species traits. Ecology Letters, 15, 1378–1386. 22938190 10.1111/j.1461-0248.2012.01861.x

[ece37808-bib-0023] de Weerdt, W. H. (1981). Transplantation experiments with Caribbean Millepora species (Hydrozoa, Coelenterata), including some ecological observations on growth forms. Bijdragen Tot De Dierkunde, 51(1), 1–19. 10.1163/26660644-05101001

[ece37808-bib-0024] Dubé, C. E. (2016). Life history of Millepora hydrocorals: New ecological and evolutionary perspectives from population genetic approaches. Doctoral dissertation, EPHE, Paris.

[ece37808-bib-0025] Eakin, C. M. , Morgan, J. A. , Heron, S. F. , Smith, T. B. , Liu, G. , Alvarez‐Filip, L. , Baca, B. , Bartels, E. , Bastidas, C. , Bouchon, C. , Brandt, M. , Bruckner, A. W. , Bunkley‐Williams, L. , Cameron, A. , Causey, B. D. , Chiappone, M. , Christensen, T. R. L. , Crabbe, M. J. C. , Day, O. , … Yusuf, Y. (2010). Caribbean corals in crisis: Record thermal stress, bleaching, and mortality in 2005. PLoS One, 5, e13969. 10.1371/journal.pone.0013969 21125021 PMC2981599

[ece37808-bib-0026] Edmunds, P. J. , & Elahi, R. (2007). The demographics of a 15‐year decline in cover of the Caribbean reef coral *Montastraea annularis* . Ecological Monographs, 77, 3–18.

[ece37808-bib-0027] Estrada‐Saldívar, N. , Jordán‐Dalhgren, E. , Rodríguez‐Martínez, R. E. , Perry, C. , & Alvarez‐Filip, L. (2019). Functional consequences of the long‐term decline of reef‐building corals in the Caribbean: Evidence of across‐reef functional convergence. Royal Society Open Science, 6(10), 190298. 10.1098/rsos.190298 PMC6837220 31824686

[ece37808-bib-0028] Fabricius, K. E. (2005). Effects of terrestrial runoff on the ecology of corals and coral reefs: Review and synthesis. Marine Pollution Bulletin, 50, 125–146. 10.1016/j.marpolbul.2004.11.028 15737355

[ece37808-bib-0029] Gardner, T. A. , Côté, I. M. , Gill, J. A. , Grant, A. , & Watkinson, A. R. (2003). Long‐term region‐wide declines in Caribbean corals. Science, 301, 958–960. 10.1126/science.1086050 12869698

[ece37808-bib-0030] Gates, R. T. (1990). Seawater temperature and sublethal coral bleaching in Jamaica. Coral Reefs, 8, 193–197. 10.1007/BF00265010

[ece37808-bib-0031] Geister, J. (1977). The influence of wave exposure on the ecological zonation of Caribbean coral reefs. Proc. Third International Coral Reef Symposium 1 23–29.

[ece37808-bib-0032] Gladfelter, W. B. (1982). White‐band disease in *Acropora palmata*: Implications for the structure and growth of shallow reefs. Bulletin of Marine Science, 32, 639–643.

[ece37808-bib-0033] Glynn, P. W. (1993). Coral reef bleaching: Ecological perspectives. Coral Reefs, 12, 1–17. 10.1007/BF00303779

[ece37808-bib-0034] González‐Barrios, F. J. , Cabral‐Tena, R. A. , & Alvarez‐Filip, L. (2021). Recovery disparity between coral cover and the physical functionality of reefs with impaired coral assemblages. Global Change Biology, 27(3), 640–651. 10.1111/gcb.15431 33131196

[ece37808-bib-0035] Green, D. H. , Edmunds, P. J. , & Carpenter, R. C. (2008). Increasing relative abundance of *Porites astreoides* on Caribbean reefs mediated by an overall decline in coral cover. Marine Ecology Progress Series, 359, 1–10.

[ece37808-bib-0036] Grime, J. P. (1977). Evidence for the existence of three primary strategies in plants and its relevance to ecological and evolutionary theory. The American Naturalist, 111, 1169–1194.

[ece37808-bib-0037] Grime, J. P. (2006). Plant strategies, vegetation processes, and ecosystem properties. John Wiley & Sons.

[ece37808-bib-0038] Hardt, M. J. (2007). Human impacts on Caribbean coral reef ecosystems. Doctoral dissertation, UC San Diego, San Diego.

[ece37808-bib-0039] Harvell, D. , Jordán‐Dahlgren, E. , Merkel, S. , Rosenberg, E. , Raymundo, L. , Smith, G. , Weil, E. , & Willis, B. (2007). Coral disease, environmental drivers, and the balance between coral and microbial associates. Oceanography, 20, 172–195. 10.5670/oceanog.2007.91

[ece37808-bib-0040] Hongo, C. (2012). Holocene key coral species in the Northwest Pacific: Indicators of reef formation and reef ecosystem responses to global climate change and anthropogenic stresses in the near future. Quaternary Science Reviews, 35, 82–99. 10.1016/j.quascirev.2012.01.011

[ece37808-bib-0041] Hughes, T. P. (1985). Life histories and population dynamics of early successional corals. 5th International Coral Reef Congress, 101–106

[ece37808-bib-0042] Hughes, T. P. , Anderson, K. D. , Connolly, S. R. , Heron, S. F. , Kerry, J. T. , Lough, J. M. , Baird, A. H. , Baum, J. K. , Berumen, M. L. , Bridge, T. C. , Claar, D. C. , Eakin, C. M. , Gilmour, J. P. , Graham, N. A. J. , Harrison, H. , Hobbs, J.‐P. , Hoey, A. S. , Hoogenboom, M. , Lowe, R. J. , … Wilson, S. K. (2018). Spatial and temporal patterns of mass bleaching of corals in the Anthropocene. Science, 359, 80–83. 10.1126/science.aan8048 29302011

[ece37808-bib-0043] Hughes, T. P. , & Jackson, J. B. C. (1985). Population dynamics and life histories of foliaceous corals. Ecological Monographs, 55, 141–166. 10.2307/1942555

[ece37808-bib-0044] Hughes, T. P. , Rodrigues, M. J. , Bellwood, D. R. , Ceccarelli, D. , Hoegh‐Guldberg, O. , McCook, L. , Moltschaniwskyj, N. , Pratchett, M. S. , Steneck, R. S. , & Willis, B. (2007). Phase shifts, herbivory, and the resilience of coral reefs to climate change. Current Biology, 17, 360–365. 17291763 10.1016/j.cub.2006.12.049

[ece37808-bib-0045] Hughes, T. P. , & Tanner, J. E. (2000). Recruitment failure, life histories, and long‐term decline of Caribbean corals. Ecology, 81 (8), 2250–2263.

[ece37808-bib-0046] Jaccard, P. (1912). The distribution of the flora in the alpine zone. New Phytologist, 11, 37–50.

[ece37808-bib-0047] Jackson, J. B. C. (1992). Pleistocene perspectives on coral reef community structure. American Zoologist, 32, 719–731.

[ece37808-bib-0048] Jackson, J. B. C. (1997). Reefs since Columbus. Coral Reefs, 16, S23–S32. 10.1007/s003380050238

[ece37808-bib-0049] Jackson, J. B. C. , Kirby, M. X. , Berger, W. H., , Bjorndal, K. A. , Botsford, L. W. , Bourque, B. J. , Bradbury, R. H. , Cooke, R. , Erlandson, J. , Estes, J. A. , Hughes, T. P. , Kidwell, S. , Lange, C. B. , Lenihan, H. S. , Pandolfi, J. M. , Peterson, C. H. , Steneck, R. S. , Tegner, M. J. ,& WarnerWarner, R. R. (2001). Historical overfishing and the recent collapse of coastal ecosystems. Science, 292, 629–637. 10.1126/science.1059199 11474098

[ece37808-bib-0050] Jackson, J. B. C. , Donovan, M. K. , Cramer, K. L. , & Lam, V. V. (2014). Status and trends of Caribbean coral reefs: 1970–2012. Global Coral Reef Monitoring Network, IUCN, Gland, Switzerland.

[ece37808-bib-0051] Jameson, S. C. , & Cairns, S. D. (2012). Neotypes for *Porites porites* (Pallas, 1766) and *Porites divaricata* Le Sueur, 1820 and remarks on other western Atlantic species of Porites (Anthozoa: Scleractinia). Proceedings of the Biological Society of Washington, 125(2), 189–207.

[ece37808-bib-0052] Khan, N. S. , Ashe, E. , Horton, B. P. , Dutton, A. , Kopp, R. E. , Brocard, G. , Engelhart, S. E. , Hill, D. F. , Peltier, W. R. , Vane, C. H. , & Scatena, F. N. (2017). Drivers of Holocene sea‐level change in the Caribbean. Quaternary Science Reviews, 155, 13–36.

[ece37808-bib-0053] Knowlton, N. (2001). The future of coral reefs. Proceedings of the National Academy of Sciences of the United States of America, 98, 5419–5425. 10.1073/pnas.091092998 11344288 PMC33228

[ece37808-bib-0054] Knowlton, N. , Weil, E. , Weigt, L. A. , & Guzman, H. M. (1992). Sibling species in *Montastraea annularis*, coral bleaching, and the coral climate record. Science, 255, 330–333. 10.1126/science.255.5042.330 17779583

[ece37808-bib-0055] Knutson, T. R. , Delworth, T. L. , Dixon, K. W. , Held, I. M. , Lu, J. , Ramaswamy, V. , Schwarzkopf, M. D. , Stenchikov, G. , & Stouffer, R. J. (2006). Assessment of twentieth‐century regional surface temperature trends using the GFDL CM2 coupled models. Journal of Climate, 19, 1624–1651. 10.1175/JCLI3709.1

[ece37808-bib-0056] Lang, J. (1973). Interspecific aggression by scleractinian corals. 2. Why the race is not only to the swift. Bulletin of Marine Science, 23, 260–279.

[ece37808-bib-0057] Lapointe, B. E. , Brewton, R. A. , Herren, L. W. , Porter, J. W. , & Hu, C. (2019). Nitrogen enrichment, altered stoichiometry, and coral reef decline at Looe Key, Florida Keys, USA: A 3‐decade study. Marine Biology, 166, 108. 10.1007/s00227-019-3538-9

[ece37808-bib-0058] Lasker, H. R. , Peters, E. C. , & Coffroth, M. A. (1984). Bleaching of reef coelenterates in the San Blas Islands, Panama. Coral Reefs, 3, 183–190. 10.1007/BF00288253

[ece37808-bib-0059] Lessios, H. A. , Cubit, J. D. , Robertson, D. R. , Shulman, M. J. , Parker, M. R. , Garrity, S. D. , & Levings, S. C. (1984). Mass mortality of *Diadema antillarum* on the Caribbean coast of Panama. Coral Reefs, 3, 173–182. 10.1007/BF00288252

[ece37808-bib-0060] Lessios, H. A. , Robertson, D. R. , & Cubit, J. D. (1984). Spread of *Diadema* mass mortality through the Caribbean. Science, 226, 335–337. 10.1126/science.226.4672.335 17749884

[ece37808-bib-0061] Lewis, J. B. (1984). The Acropora inheritance: A reinterpretation of the development of fringing reefs in Barbados, West Indies. Coral Reefs, 3, 117–122. 10.1007/BF00301955

[ece37808-bib-0062] Loya, Y. (1976). Effects of water turbidity and sedimentation on the community structure of Puerto Rican corals. Bulletin of Marine Science, 26(4), 450–466.

[ece37808-bib-0063] Loya, Y. , Sakai, K. , Yamazato, K. , Nakano, Y. , Sambali, H. , & van Woesik, R. (2001). Coral bleaching: The winners and the losers. Ecology Letters, 4, 122–131. 10.1046/j.1461-0248.2001.00203.x

[ece37808-bib-0064] MacKenzie, D. I. , Nichols, J. D. , Sutton, N. , Kawanishi, K. , & Bailey, L. L. (2005). Improving inferences in population studies of rare species that are detected imperfectly. Ecology, 86, 1101–1113. 10.1890/04-1060

[ece37808-bib-0065] MacNeil, M. A. , Mellin, C. , Matthews, S. , Wolff, N. H. , McClanahan, T. R. , Devlin, M. , Drovandi, C. , Mengersen, K. , & Graham, N. A. (2019). Water quality mediates resilience on the Great Barrier Reef. Nature Ecology and Evolution, 3, 620–627. 30858590 10.1038/s41559-019-0832-3

[ece37808-bib-0066] McClanahan, T. R. , & Muthiga, N. A. (1998). An ecological shift in a remote coral atoll of Belize over 25 years. Environmental Conservation, 25, 122–130.

[ece37808-bib-0067] Mellin, C. , Aaron MacNeil, M. , Cheal, A. J. , Emslie, M. J. , & Julian Caley, M. (2016). Marine protected areas increase resilience among coral reef communities. Ecology Letters, 19, 629–637. 10.1111/ele.12598 27038889

[ece37808-bib-0068] Mesolella, K. J. (1967). Zonation of uplifted Pleistocene coral reefs on Barbados, West Indies. Science, 156(3775), 638–640. 17837159 10.1126/science.156.3775.638

[ece37808-bib-0069] Milliman, J. D. (1969). Four southwestern Caribbean atolls: Courtown Cays, Albuquerque Cays, Roncador Bank and Serrana Bank. Atoll Research Bulletin, 129, 1–46. 10.5479/si.00775630.129.1

[ece37808-bib-0070] Paddack, M. J. , Reynolds, J. D. , Aguilar, C. , Appeldoorn, R. S. , Beets, J. , Burkett, E. W. , Chittaro, P. M. , Clarke, K. , Esteves, R. , Fonseca, A. C. , Forrester, G. E. , Friedlander, A. M. , García‐Sais, J. , González‐Sansón, G. , Jordan, L. K. B. , McClellan, D. B. , Miller, M. W. , Molloy, P. P. , Mumby, P. J. , Nagelkerken, I. , … Côté, I. M. (2009). Recent region‐wide declines in Caribbean reef fish abundance. Current Biology, 19, 590–595. 19303296 10.1016/j.cub.2009.02.041

[ece37808-bib-0071] Pandolfi, J. M. , Bradbury, R. H. , Sala, E. , Hughes, T. P. , Bjorndal, K. A. , Cooke, R. G. , McArdle, D. , McClenachan, L. , Newman, M. J. H. , Paredes, G. , Warner, R. R. , & Jackson, J. B. C. (2003). Global trajectories of the long‐term decline of coral reef ecosystems. Science, 301(5635), 955–958. 12920296 10.1126/science.1085706

[ece37808-bib-0072] Pandolfi, J. M. , & Jackson, J. B. (2001). Community structure of Pleistocene coral reefs of Curaçao, Netherlands Antilles. Ecological Monographs, 71, 49–67.

[ece37808-bib-0073] Pandolfi, J. M. , & Jackson, J. B. (2006). Ecological persistence interrupted in Caribbean coral reefs. Ecology Letters, 9, 818–826. 16796572 10.1111/j.1461-0248.2006.00933.x

[ece37808-bib-0075] Perry, C. T. , Steneck, R. S. , Murphy, G. N. , Kench, P. S. , Edinger, E. N. , Smithers, S. G. , & Mumby, P. J. (2014). Regional‐scale dominance of non‐framework building corals on Caribbean reefs affects carbonate production and future reef growth. Global Change Biology. 21(3), 1153–1164. 10.1111/gcb.12792 25537577

[ece37808-bib-0076] Precht, W. F. , Gintert, B. E. , Robbart, M. L. , Fura, R. , & van Woesik, R. (2016). Unprecedented disease‐related coral mortality in Southeastern Florida. Scientific Reports, 6, 31374. 10.1038/srep31374 27506875 PMC4979204

[ece37808-bib-0077] R Core Team (2018). R: A language and environment for statistical computing. R Foundation for Statistical Computing.

[ece37808-bib-0078] Randall, C. J. , & Szmant, A. M. (2009). Elevated temperature affects development, survivorship, and settlement of the elkhorn coral, *Acropora palmata* (Lamarck 1816). The Biological Bulletin, 217(3), 269–282. 20040751 10.1086/BBLv217n3p269

[ece37808-bib-0079] Randall, C. J. , & van Woesik, R. (2015). Contemporary white‐band disease in Caribbean corals driven by climate change. Nature Climate Change, 5, 375–379.

[ece37808-bib-0080] Randall, J. E. (1961). Overgrazing of algae by herbivorous marine fishes. Ecology, 42, 812–812. 10.2307/1933510

[ece37808-bib-0081] Richardson, L. E. , Graham, N. A. , Pratchett, M. S. , Eurich, J. G. , & Hoey, A. S. (2018). Mass coral bleaching causes biotic homogenization of reef fish assemblages. Global Change Biology, 24, 3117–3129. 10.1111/gcb.14119 29633512

[ece37808-bib-0082] Rogers, A. , Blanchard, J. L. , & Mumby, P. J. (2014). Vulnerability of coral reef fisheries to a loss of structural complexity. Current Biology, 24(9), 1000–1005. 10.1016/j.cub.2014.03.026 24746794

[ece37808-bib-0083] Rutzler, K. , & Macintyre, I. G. (1982). The habitat distribution and community structure of the barrier reef complex at Carrier Bow Cay, Belize. In K. Rutzler , & I. G. Macintyre (Eds.), The Atlantic Barrier Reef ecosystem at Carrie Bow Cay, Belize (pp. 9–45). Smithsonian.

[ece37808-bib-0084] Sheppard, C. , & Rioja‐Nieto, R. (2005). Sea surface temperature 1871–2099 in 38 cells in the Caribbean region. Marine Environment Research, 60, 389–396. 10.1016/j.marenvres.2004.12.006 15769506

[ece37808-bib-0085] Smith, S. R. (1992). Patterns of coral recruitment and post‐settlement mortality on Bermuda's reefs: Comparisons to Caribbean and Pacific reefs. American Zoologist, 32, 663–673. 10.1093/icb/32.6.663

[ece37808-bib-0086] Steneck, R. , Arnold, S. N. , Boenish, R. , De Leon, R. , Mumby, P. J. , Rasher, D. B. , & Wilson, M. (2019). Managing recovery resilience in coral reefs against climate‐induced bleaching and hurricanes: A 15 year case study from Bonaire, Dutch Caribbean. Frontiers in Marine Science, 6, 265. 10.3389/fmars.2019.00265

[ece37808-bib-0087] van Woesik, R. , & Randall, C. J. (2017). Coral disease hotspots in the Caribbean. Ecosphere, 8, e01814. 10.1002/ecs2.1814

[ece37808-bib-0088] Vega Thurber, R. L. , Mydlarz, L. D. , Brandt, M. , Harvell, D. , Weil, E. , Raymundo, L. , Willis, B. L. , Langevin, S. , Tracy, A. M. , Littman, R. , Kemp, K. M. , Dawkins, P. , Prager, K. C. , Garren, M. , & Lamb, J. (2020). Deciphering coral disease dynamics: Integrating host, microbiome, and the changing environment. Frontiers in Ecology and Evolution, 8, 402.

[ece37808-bib-0089] Walton, C. , Hayes, N. K. , & Gilliam, D. S. (2018). Impacts of a regional, multi‐year, multi‐species coral disease outbreak in Southeast Florida. Frontiers in Marine Science, 5, 323. 10.3389/fmars.2018.00323

[ece37808-bib-0090] Weber, D. , Hinterman, U. , & Zangger, A. (2004). Scale and trends in species richness: Considerations for monitoring biological diversity for political purposes. Global Ecological Biogeography, 13, 97–104. 10.1111/j.1466-882X.2004.00078.x

[ece37808-bib-0091] Weil, E. , Cróquer, A. , & Urreiztieta, I. (2009). Yellow band disease compromises the reproductive output of the Caribbean reef‐building coral *Montastraea faveolata* (Anthozoa, Scleractinia). Diseases of Aquatic Organisms, 87, 45–55. 20095240 10.3354/dao02103

[ece37808-bib-0092] Wiedenmann, J. , D’Angelo, C. , Smith, E. G. , Hunt, A. N. , Legirt, F. E. , Postle, A. D. , & Achterberg, E. P. (2013). Nutrient enrichment can increase the susceptibility of reef corals to bleaching. Nature Climate Change, 3, 160–164.

